# Ergodic Rate for Fading Interference Channels with Proper and Improper Gaussian Signaling

**DOI:** 10.3390/e21100922

**Published:** 2019-09-23

**Authors:** Mohammad Soleymani, Ignacio Santamaria, Christian Lameiro, Peter J. Schreier

**Affiliations:** 1 Signal and System Theory Group, Universität Paderborn, 33098 Paderborn, Germany; christian.lameiro@sst.upb.de (C.L.); peter.schreier@sst.upb.de (P.J.S.); 2 Department of Communications Engineering, University of Cantabria, 39005 Santander, Cantabria, Spain; i.santamaria@unican.es

**Keywords:** achievable ergodic rate region, fading channels, improper Gaussian signaling, 2-user interference channel

## Abstract

This paper studies the performance of improper Gaussian signaling (IGS) over a 2-user Rayleigh single-input single-output (SISO) interference channel, treating interference as noise. We assume that the receivers have perfect channel state information (CSI), while the transmitters have access to only statistical CSI. Under these assumptions, we consider a signaling scheme, which we refer to as proper/improper Gaussian signaling or PGS/IGS, where at most one user may employ IGS. For the Rayleigh fading channel model, we characterize the statistical distribution of the signal-to-interference-plus-noise ratio at each receiver and derive closed-form expressions for the ergodic rates. By adapting the powers, we characterize the Pareto boundary of the ergodic rate region for the 2-user fading IC. The ergodic transmission rates can be attained using fixed-rate codebooks and no optimization is involved. Our results show that, in the moderate and strong interference regimes, the proposed PGS/IGS scheme improves the performance with respect to the PGS scheme. Additionally, we numerically compute the ergodic rate region of the full IGS scheme when both users can employ IGS and their transmission parameters are optimized by an exhaustive search. Our results suggest that most of the Pareto optimal points for the 2-user fading IC channel are attained when either both users transmit PGS or when one transmits PGS and the other transmits maximally improper Gaussian signals and time sharing is allowed.

## 1. Introduction

Interference management techniques play a key role in modern wireless communication systems, which are mostly interference-limited [1]. The capacity-achieving signaling in point-to-point communication systems is proper Gaussian signaling (PGS) [2]. Nevertheless, this is not generally the case in interference-limited systems, where it has been shown that improper (or asymmetric) Gaussian signaling (IGS) can improve the performance of various interference-limited systems [3,4,5,6,7,8,9,10,11,12,13,14,15,16,17,18,19,20,21]. In contrast to proper signals, the real and imaginary parts of an improper signal are correlated and/or have unequal powers [22,23].

### 1.1. Related Work

The Gaussian interference channel (IC) is a fundamental communication scenario where multiple users share the same frequency and time resources for the transmission of the desired messages. It has been vastly studied over the last decades [24,25,26,27,28,29,30,31] but its capacity is still unknown in the general case [24,25,26]. In some particular cases such as the very strong and very weak interference regimes, the capacity-achieving schemes are known [26]. For example, in the very strong interference regime, the capacity of the 2-user IC is achieved by successive interference cancellation (SIC), in which the interfering signals are firstly decoded and then canceled from the received signal [26,27,28]. In other words, in the strong interference regime, the interference has to be decoded in order to achieve capacity. Obviously, SIC is not a linear operation and has a high computational cost, specially in a multiuser setting. On the other hand, when the interference level is very low, which is also referred as the very weak interference regime, treating interference as noise (TIN) is the capacity achieving strategy [25,26]. TIN is a simple and practical decoding strategy, which turns out to be optimal from generalized degree of freedom (GDoF) point of view in low interference regimes [31].

In summary and at a coarse level, if the interference is strong, the optimal interference management approach is to decode it along with the desired signal, whereas if the interference is weak, then the interference management approach is to treat interference as noise. If the strength of the interference is comparable to the strength of the desired signal, the degrees-of-freedom (DoF) optimal interference management approach is to apply interference alignment (IA) [32,33,34,35,36]. IA designs transmit precoders that reduce the dimension of the interference subspace, in such a way that the interference can be zero-forced at each receiver thus getting an interference-free subspace for the transmission of the desired signals. However, the feasibility of IA for channels with constant coefficients depends on the number of antennas and hence packing the interference into a low-dimensional subspace is not always possible. Hence, in some practical scenarios, we have to tolerate and manage interference to improve the system performance. As a result, in modern wireless communication systems non-orthogonal multiple access (NOMA) schemes play an essential role [37,38,39].

Another approach to handle interference is to employ IGS, which has been considered as an interference-management technique for the first time in Reference [3]. The authors of Reference [3] showed that IGS can increase the degrees-of-freedom (DoF) of the 3-user single-input single-output (SISO) interference channel (IC) with constant channel coefficients. In Reference [4], this result was extended to the 4-user SISO IC and, in Reference [5], the authors showed that IGS can also increase the DoF of the 2-user multiple-input, multiple-output (MIMO) X channel. In these papers, it is shown that IGS with IA can improve the DoF of different interference-limited systems. However, IGS can also be beneficial from rate and/or power/energy-efficiency point of view when interference is treated as noise [6,7,8,9,10,11,12,13,14,15,16,17,18,19,20,21]. It was shown in Reference [6], for example, that the secondary user (SU) in an underlay cognitive radio (UCR) system may increase its rate by transmitting IGS codewords while satisfying the rate requirement of the primary user (PU). The superiority of IGS over PGS schemes for the Z-IC was also established in several works [7,8,9] and similar performance improvements have also been shown for the 2-user IC in References [10,11,12]. The benefits of IGS from Energy efficiency perspective were also studied in different scenarios in References [15,16].

All the aforementioned works [3,4,5,6,7,8,9,10,11,12,13,14,15,16,17] assume perfect and instantaneous channel state information at the transmitter side (CSIT) and they additionally involve some optimization of the parameters of the transmitted improper signal (e.g., circularity coefficient and transmit power). However, perfect CSIT is a restrictive assumption in practice.

The number of works studying the performance of IGS in scenarios with either imperfect or statistical CSIT in fading channels is much smaller. For instance, in Reference [18], we proposed a robust IGS for a 2-user IC with imperfect instantaneous CSIT and showed that IGS can enlarge the achievable rate region and outperform PGS. Reference [19] considers an underlay cognitive radio (UCR) network in which the secondary user has only statistical CSIT and optimizes its transmit power and degree of impropriety to maintain a given primary user (PU) outage probability. A more challenging UCR scenario where the PU is assumed to work using full-duplex mode is considered in Reference [20] for Rayleigh fading channels. Nevertheless, References [19,20] only consider block fading models and the probability of outage as the figure of merit to optimize. In Reference [21], the authors studied a full-duplex relay system over Nakagami-*m* fading channels. They derived the outage probability for a given target rate as well as the ergodic rate of the system and showed the benefits of IGS.

### 1.2. Contribution

In this work, we study the achievable ergodic rates of IGS schemes over the 2-user fading IC when interference is treated as noise. To the best of our knowledge, it is the first work that analyzes the ergodic rate of improper signaling in the 2-user IC. For the case where at most one user employs IGS (denoted as PGS/IGS scheme), we derive closed-form expressions for the ergodic rates for both users. These rates can be attained using fixed-rate codebooks and no optimization is involved. For the more general IGS/IGS scheme where both users can employ improper signaling no closed-form expressions for the rates exist and hence we compute numerically the optimal transmission parameters and the boundary of the rate region. In our model, the users have perfect and instantaneous CSI at the receiver side (CSIR) but they only have access to statistical CSIT. In other words, the transmitters know the parameters defining the Rayleigh fading distributions of the direct and interfering links. Note that the perfect instantaneous CSIR can be achieved using, for example, training sequences [18,40]. Under these assumptions, we derive the achievable ergodic rates when the users transmit proper and improper Gaussian codewords.

The main contributions of this paper are as follows:

- We derive closed-form expressions for the achievable ergodic rate of the PGS and IGS users. We also characterize the achievable ergodic rate region of the mixed PGS/IGS scheme by allowing power control.
- Our proposed scheme incurs no additional overhead at the transmitter side when both users transmit at maximum power. Moreover, in order to operate at a specific Pareto-optimal point, the transmitters (or a central entity implementing the power control module) should be informed about the average gain of the direct and interference links, which are scalar values and can be easily sent to the transmitters. Hence, the PGS/IGS scheme is simple to implement in practice.
- Through numerical examples, we show that our proposed PGS/IGS scheme substantially outperforms the PGS scheme in moderate and strong interference regimes. Moreover, we analyze the maximum sum-rate point for the symmetric 2-user IC when both users transmit at maximum power. Under this condition, the sum-rate point is attained either with PGS or with PGS/MIGS (maximally IGS); that is, one user transmits proper signals while the other user employs maximally improper signaling. Then, the maximum sum-rate point does not require any optimization and/or power control.
- We also compare our results with the more general scheme where both users can employ IGS and their transmission parameters are numerically optimized. Our numerical results show that the union of the PGS and PGS/MIGS schemes with time sharing attains almost the whole Pareto-optimal points in the rate region and little is gained by allowing both users to employ IGS.

### 1.3. Paper Outline

The rest of the paper is organized as follows. In Section 2, we define the system model parameters and formulate the problem. In Section 3 we propose our scheme and derive the ergodic rates of the PGS/IGS scheme. Section 4 presents some numerical examples and the main conclusions are summarized in Section 5.

## 2. Signal Model and Problem Statement

### 2.1. Notation and Preliminaries

We denote the probability density function (PDF) of a zero-mean complex Gaussian distribution with variance $p_{x}=E\left\{{\left|X\right|}^{2}\right\}$ and complementary variance $q_{x}=E\left\{X^{2}\right\}$ as $X∼\mathrm{CN}(0,p_{x},q_{x})$. We define the circularity coefficient of *X* as $\kappa_{x}=\frac{|q_{x}|}{p_{x}}$, where $0\leq \kappa_{x}\leq 1$ [22,23]. The variable *X* is proper if $\kappa_{x}=0$. Otherwise, *X* is improper (a.k.a. asymmetric or non-circular). Moreover, we call *X* maximally improper if $\kappa_{x}=1$.

A random variable *X* that is Gamma-distributed with shape parameter α and rate parameter β is denoted as X∼Γ(α,β). Its PDF is $f_{X}\left(x\right)=\frac{\beta^{\alpha }x^{\alpha -1}}{\Gamma (\alpha )}e^{-\beta x}$, x≥0, where Γ(α) is the gamma function. If X∼Γ(1,β), then *X* has an exponential distribution.

### 2.2. Signal Model

**Figure 1:**
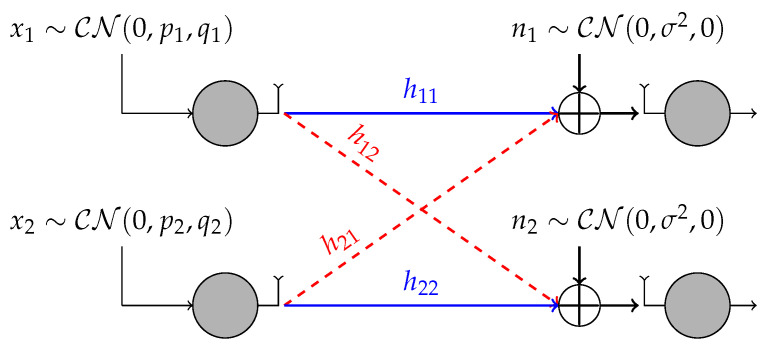
The 2-user interference channel (IC) with proper/improper Gaussian signaling.

We consider the 2-user SISO IC depicted in Figure 1. The received signal at receiver i∈{1,2} is

(1) $$y_{i}=h_{1i}x_{1}+h_{2i}x_{2}+n_{i},$$

where $x_{i}$, $n_{i}$ and $h_{ji}$ for j∈{1,2} are, respectively, the transmit signal of user *i*, proper Gaussian noise with variance $\sigma^{2}$ and the channel coefficient for the link between transmitter *j* and receiver *i*. We consider Rayleigh fading channels modeled as

(2) $$\begin{array}{cc} h_{ii} & ∼\mathrm{CN}(0,\sqrt{{\mathrm{snr}}_{i}},0), \end{array}$$

(3) $$\begin{array}{cc} h_{\bar{ı}i} & ∼\mathrm{CN}(0,\sqrt{{\mathrm{inr}}_{i}},0), \end{array}$$

where ${\mathrm{snr}}_{i}$ and ${\mathrm{inr}}_{i}$ for $i,\bar{ı}\in \left\{1,2\right\}$ and $i\neq \bar{ı}$ are, respectively, the average gain of the direct and interference links of receiver *i*. Additionally, the channel gains are Gamma distributed, that is,

(4) $$\begin{array}{cc} |h_{ii}{|}^{2} & ∼\Gamma (1,1/{\mathrm{snr}}_{i}), \end{array}$$

(5) $$\begin{array}{cc} |h_{\bar{ı}i}{|}^{2} & ∼\Gamma (1,1/{\mathrm{inr}}_{i}). \end{array}$$

We assume that the transmitters know neither the fading states on which they transmit, nor the fading states of the interfering signal. On the other hand, we assume that the receivers have perfect knowledge of the channels.

We treat interference as noise and assume that users may employ IGS. Thus, the instantaneous rate of user *i* is [18]

(6) $$R_{i}\;=\;\frac{1}{2}\;\log_{2}\;\left(\;\frac{(\sigma^{2}\;+p_{1}|h_{1i}{|}^{2}\;+p_{2}|h_{2i}{|}^{2}{)}^{2}\;-{\left|q_{1}h_{1i}^{2}+q_{2}h_{2i}^{2}\right|}^{2}}{(\sigma^{2}+p_{\bar{ı}}|h_{\bar{ı}i}{|}^{2}{)}^{2}-{\left|q_{\bar{ı}}h_{\bar{ı}i}^{2}\right|}^{2}}\;\right)\;,$$

where $p_{i}$ and $q_{i}$ are the transmission power and complementary variance of user *i*, respectively. Hereafter, we consider $\sigma^{2}=1$ without loss of generality. Moreover, the power budget of user *i* is $P_{i}$.

We first consider the scenario, where at most one user may employ IGS. For notational simplicity in this section, we consider user 1 as the PGS user without loss of generality. Obviously, there is no difference between the users and either user can be the PGS user. The instantaneous rate of the PGS user is

(7) $$\begin{array}{c} R_{1}^{\mathrm{PGS}}=\frac{1}{2}\log_{2}\left(1+\frac{X_{1}}{1+\left(1+\kappa \right)Y_{1}}\right)+\frac{1}{2}\log_{2}\left(1+\frac{X_{1}}{1+\left(1-\kappa \right)Y_{1}}\right), \end{array}$$

where $X_{i}=p_{i}{\left|h_{ii}\right|}^{2}∼\Gamma \left(1,1/\left(p_{i}{\mathrm{snr}}_{i}\right)\right)$ and $Y_{i}=p_{\bar{ı}}{\left|h_{\bar{ı}i}\right|}^{2}∼\Gamma \left(1,1/\left(p_{\bar{ı}}{\mathrm{inr}}_{i}\right)\right)$ are independent Gamma distributed random variables and κ is the circularity coefficient of the IGS user. The instantaneous rate of the IGS user is

(8) $$\begin{array}{c} R_{2}^{\mathrm{IGS}}\;=\;\frac{1}{2}\log_{2}\left(\;1\;+\frac{\left(1\;+\kappa \right)X_{2}}{1+Y_{2}}\;\right)\;+\;\frac{1}{2}\log_{2}\left(\;1\;+\frac{\left(1-\kappa \right)X_{2}}{1+Y_{2}}\;\right)\;. \end{array}$$

A special case of this scheme is MIGS, that is, κ=1. In this case, the rate of the PGS user simplifies to

(9) $$R_{1}^{\mathrm{PGS}}=\underset{\mathrm{interference}\;\mathrm{free}}{\underbrace{\frac{1}{2}\log_{2}\left(1+X_{1}\right)}}+\underset{\mathrm{with}\;\mathrm{interference}}{\underbrace{\frac{1}{2}\log_{2}\left(1+\frac{X_{1}}{1+2Y_{1}}\right)}}.$$

Since the MIGS user transmits only on one dimension, there is an interference-free dimension for the PGS user. We can exploit this interference-free dimension even without CSIT due to the fact that the receivers know the channels perfectly. Additionally, the rate of the MIGS user is

(10) $$R_{2}^{\mathrm{MIGS}}=\frac{1}{2}\;\log_{2}\;\;\left(1+\frac{2X_{2}}{1+Y_{2}}\right).$$

As we observe in Equation (10), the PGS user causes less interference to the MIGS user because both the PGS user and the noise divide their power between the two dimensions. The downside is that the MIGS user loses some rate because only one signal dimension is exploited for transmission. However, the MIGS user can compensate for this rate loss by increasing its transmission power if the power budget has not been fully used and the overall effect can be beneficial in terms of sum-rate. As indicated, the PGS user has an interference-free dimension, which ensures a minimum instantaneous achievable rate of

(11) $$R_{1}^{\mathrm{PGS}}>\frac{1}{2}\log_{2}\left(1+X_{1}\right).$$

### 2.3. Problem Statement

In this paper, we derive the achievable ergodic rates for the PGS/IGS scheme under fast fading channels (i.e., when the codewords span a large number of fading states). The achievable ergodic rate is defined as the expected achievable rate $\bar{R}=E\left\{R\right\}$. In order to derive the achievable ergodic rate region, we employ the Pareto boundary, which is defined in Definition 1.

**Definition** **1** ([7]). *The ergodic rate pair (${\bar{R}}_{1},{\bar{R}}_{2}$) is called Pareto-optimal if (${\bar{R}}_{1}^{\prime },{\bar{R}}_{2}$) and (${\bar{R}}_{1},{\bar{R}}_{2}^{\prime }$), with ${\bar{R}}_{1}^{\prime }>{\bar{R}}_{1}$ and ${\bar{R}}_{2}^{\prime }>{\bar{R}}_{2}$, are not achievable.*

The whole achievable ergodic rate region can be obtained by varying the powers and complementary variances of the transmit symbols. In References [12,41] it is proven that the Pareto-optimal rates for the 2-user IC when interference is treated as noise are obtained when at least one user transmits at maximum power. This result, which was originally proved for the case where the transmitters have perfect CSIT, is also valid for our ergodic fast fading channel model.

## 3. PGS/IGS Scheme

In this section, our goal is to derive the achievable pair of ergodic rates $\left({\bar{R}}_{1}^{\mathrm{PGS}},{\bar{R}}_{2}^{\mathrm{IGS}}\right)$ obtained by taking the mathematical expectation of $R_{1}^{\mathrm{PGS}}$ in Equation (7) and $R_{2}^{\mathrm{IGS}}$ in Equation (8). We first present Lemma 1 in order to derive the ergodic rate of the users. Then we present the achievable ergodic rates in Theorem 1.

**Lemma** **1.** *Let us define the random variable $Z=\frac{\beta X}{1+\alpha Y},$ where α≥0 and β≥0 are given constants and $X∼\Gamma (1,\frac{1}{\mathrm{snr}})$ and $Y∼\Gamma (1,\frac{1}{\mathrm{inr}})$ are independent Gamma distributed random variables. The cumulative density function (CDF) of Z is*

(12) $$F_{Z}\left(z\right)=1-\frac{e^{-\frac{z}{\beta snr}}}{1+\frac{\alpha inr}{\beta snr}z}.$$

*The expectation of Z is*

(13) $$E\left\{Z\right\}=\left\{\begin{array}{ccc} \beta snr & if & \alpha =0,\\ \left(\frac{\beta snr}{\alpha inr}\right)e^{\frac{1}{\beta snr}}E_{1}\left(\frac{1}{\beta snr}\right) & if & \alpha >0. \end{array}\right.$$

*Additionally, the expectation of R=log(1+Z) is*

(14) $$\begin{array}{c} f\left(\beta \mathrm{snr},\alpha \mathrm{inr}\right)≜\bar{R}=\;\left\{\;\begin{array}{ccc} e^{\frac{1}{\beta \mathrm{snr}}}E_{1}\left(\frac{1}{\beta \mathrm{snr}}\right) & if & \alpha =0,\\ \frac{\beta \mathrm{snr}\left(e^{\frac{1}{\beta \mathrm{snr}}}E_{1}\left(\frac{1}{\beta \mathrm{snr}}\right)-e^{\frac{1}{\alpha \mathrm{inr}}}E_{1}\left(\frac{1}{\alpha \mathrm{inr}}\right)\right)}{\beta \mathrm{snr}-\alpha \mathrm{inr}},\; & if & \beta \mathrm{snr}\neq \alpha \mathrm{inr}>0\\ e^{\frac{1}{\beta \mathrm{snr}}}E_{2}\left(\frac{1}{\beta \mathrm{snr}}\right),\; & if & \beta \mathrm{snr}=\alpha \mathrm{inr}>0 \end{array}\right. \end{array}$$

*where the $E_{n}\left(x\right)$ function is defined by the integral (for n≥1 and x>0)*

(15) $$E_{n}\left(x\right)=\int_{1}^{\infty }\frac{e^{-xt}}{t^{n}}dt.$$

**Proof of Lemma** **1.** Please refer to Appendix A. □

Note that when β=0, the variable *Z* defined in Lemma 1 is Z=0. To include this case, we define f(0,αinr)≜0. Moreover, notice that $E_{n}\left(x\right)$ can be written in terms of the upper incomplete gamma function as $E_{n}\left(x\right)=x^{n-1}\Gamma \left(1-n,x\right)$, where

(16) $$\Gamma \left(s,x\right)=\int_{x}^{\infty }t^{s-1}e^{-t}.$$

$E_{1}\left(x\right)=\Gamma \left(0,x\right)$ is the exponential integral. These functions can be computed using standard software packages.

**Theorem** **1.** *The ergodic rates of the PGS and the IGS users are, respectively,*

(17) $$\begin{array}{cc} {\bar{R}}_{1}^{PGS} & \;=\;f\left(p_{1}{\mathrm{snr}}_{1},\left(1\;+\;\kappa \right)p_{2}{\mathrm{inr}}_{1}\right)\;+\;f\left(p_{1}{\mathrm{snr}}_{1},\left(1\;-\;\kappa \right)p_{2}{\mathrm{inr}}_{1}\right)\;, \end{array}$$

(18) $$\begin{array}{cc} {\bar{R}}_{2}^{IGS} & \;=\;f\left(\left(1\;+\;\kappa \right)p_{2}{\mathrm{snr}}_{2},p_{1}{\mathrm{inr}}_{2}\right)\;+\;f\left(\left(1\;-\;\kappa \right)p_{2}{\mathrm{snr}}_{2},p_{1}{\mathrm{inr}}_{2}\right)\;, \end{array}$$

*where f(x,y) is given by Equation (14).*

**Proof of Theorem** **1.** The ergodic rate of users are given by taking expectations of Equations (7) and (8). Hence, by applying the results of Lemma 1 and substituting the corresponding α and β, we can prove the theorem. □

**Remark** **1.** *The ergodic rate of the PGS user is strictly increasing in κ, while the ergodic rate of the IGS user is strictly decreasing in κ. This is due to the fact that the instantaneous rate of the PGS (IGS) user is strictly increasing (decreasing) in κ.*

We can derive the achievable ergodic rate region by varying the transmission power of the two users. As indicated in Section 2.3, every point on the boundary of the achievable ergodic rate region is obtained when at least one user transmits with maximum power [12,41]. Thus, the whole Pareto optimal region for the PGS/IGS scheme can be derived as

$$\begin{array}{cc} S^{IGS}= & \;\;\;\;\;\;\;\;\underset{\begin{array}{c} 0\leq \kappa_{2}\leq 1\\ 0\leq p_{2}\leq P_{2} \end{array}}{⋃}\;\;\;\;\;\;\;\left({\bar{R}}_{1}^{\mathrm{PGS}}\left(P_{1},p_{2},\kappa_{2}\right),{\bar{R}}_{2}^{\mathrm{IGS}}\left(P_{1},p_{2},\kappa_{2}\right)\right)\underset{\begin{array}{c} 0\leq \kappa_{2}\leq 1\\ 0\leq p_{1}\leq P_{1} \end{array}}{⋃}\;\;\;\;\;\;\;\left({\bar{R}}_{1}^{\mathrm{PGS}}\left(p_{1},P_{2},\kappa_{2}\right),{\bar{R}}_{2}^{\mathrm{IGS}}\left(p_{1},P_{2},\kappa_{2}\right)\;\right) \end{array}$$

(19) $$\begin{array}{c} \;\;\;\;\;\;\;\;\;\;\underset{\begin{array}{c} 0\leq \kappa_{1}\leq 1\\ 0\leq p_{2}\leq P_{2} \end{array}}{⋃}\;\;\;\;\;\;\;\left({\bar{R}}_{1}^{\mathrm{IGS}}\left(P_{1},p_{2},\kappa_{1}\right),{\bar{R}}_{2}^{\mathrm{PGS}}\left(P_{1},p_{2},\kappa_{1}\right)\;\right)\underset{\begin{array}{c} 0\leq \kappa_{1}\leq 1\\ 0\leq p_{1}\leq P_{1} \end{array}}{⋃}\;\;\;\;\;\;\;\left({\bar{R}}_{1}^{\mathrm{IGS}}\left(p_{1},P_{2},\kappa_{1}\right),{\bar{R}}_{2}^{\mathrm{PGS}}\left(p_{1},P_{2},\kappa_{1}\right)\;\right)\;. \end{array}$$

Note that the achievable ergodic rate region in Equation (19) is the union of the achievable ergodic rate region of two transmission strategies:

1. User 1 employs PGS, while user 2 may employ IGS,
2. User 2 employs PGS, while user 1 may employ IGS.

## 4. Numerical Examples

We consider a 2-user fading IC where the channel coefficients of the direct links are drawn from $h_{ii}∼\mathrm{CN}\left(0,\sqrt{\mathrm{snr}},0\right)$ and the channel coefficients of the interfering links are drawn from $h_{i\bar{ı}}∼\mathrm{CN}\left(0,\sqrt{\mathrm{inr}},0\right)$. The four channel coefficients are independent random variables. In this way, the performance of the proposed schemes can be analyzed in terms of the average snr and inr, which are the same at both receivers. Moreover, we assume $P_{1}=P_{2}=1$ and $\sigma^{2}=1$. Indeed, we reflect the effect of the transmission power as well as the noise power by the gain of the direct and interference links, that is, snr and inr. Since the fading direct and interference channels are symmetric, the two users send information at the same rate.

When at most one user employs IGS, the instantaneous rates are independent of the phases of the channels. Additionally, it allows us to find closed-form expressions of the ergodic rates. However, the question remains: *How far is the PGS/IGS scheme from the Pareto-optimal design of a scheme that would allow both users to use IGS on the 2-user IC?*

To answer the question, we compare the performance of our scheme with an exhaustive search over all design parameters $\left(\kappa_{1},\kappa_{2},p_{1},p_{2}\right)$ when both users may employ IGS. Unfortunately, to the best of our knowledge, there is no closed-form solution for the ergodic rates when both users employ IGS. Thus, we estimate the ergodic rate of the 2-user IC when both users employ IGS by Monte Carlo simulations. That is, we generate a large number ($10^{5}$) of channels and compute the instantaneous rate achieved by the transmission parameters for each channel realization. Finally, we estimate the ergodic rate by averaging the instantaneous rates. Once the ergodic rate pairs have been estimated for many different transmission parameters, the Pareto boundary for the IGS/IGS scheme can readily be estimated.

The considered schemes are denoted as follows.

- **PGS**: The PGS scheme with $\kappa_{1}=\kappa_{2}=0$.
- **MIGS**: The PGS/MIGS scheme with κ=1.
- **E-IGS**: The exhaustive search for IGS when both users may employ IGS.
- **O-P/IGS**: The PGS/IGS scheme with the optimal κ.
- **MIGS-U${}_{i}$**: The PGS/MIGS scheme when the MIGS user is user *i*.
- **MIGS-TS**: The PGS/MIGS scheme with time sharing over the points with maximum power for both users.

**Figure 2:**
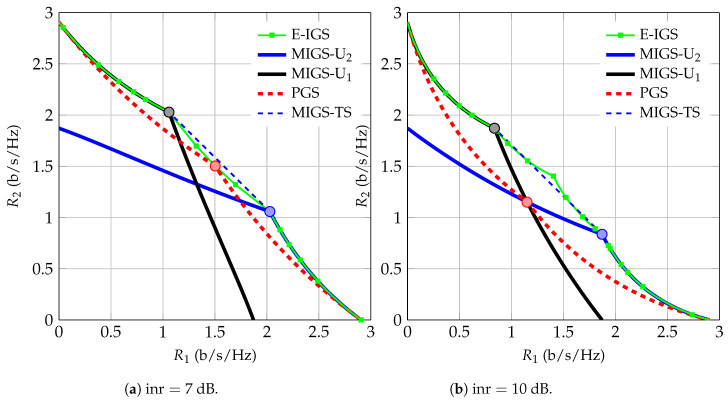
Proper Gaussian signaling (PGS) and improper Gaussian signaling (IGS) rate regions for a symmetric 2-user IC with snr=10 dB.

In Figure 2, we depict the achievable ergodic rate region of our proposed schemes for snr=10 dB. As can be observed, the union of the ergodic rate regions achievable by the PGS and MIGS schemes is very close to the optimal achievable ergodic rate region. If we employ time sharing over the points with maximum power for both users in the union of the PGS and PGS/MIGS schemes (indicated by circles in Figure 2), we attain almost the entire Pareto-optimal region of the 2-user IC when both users are allowed to employ IGS.

**Figure 3:**
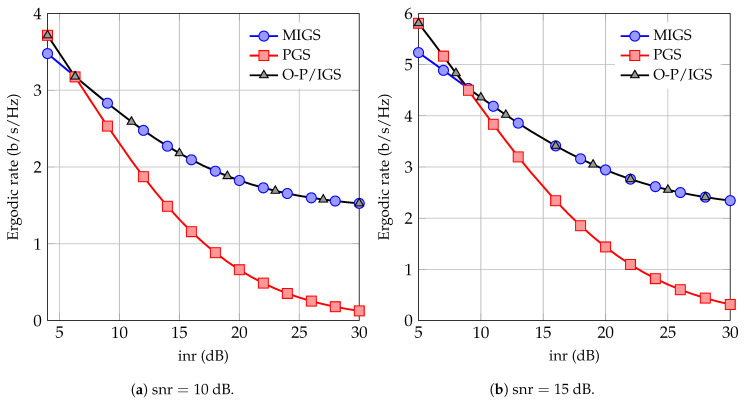
Sum ergodic rate of the schemes as a function of inr.

In Figure 3, we show the sum-ergodic rate as a function of the inr when both users transmit at maximum power. Note that, in this case, the sum-ergodic rate can be achieved by red points for the PGS scheme and by either black or blue points for the PGS/MIGS scheme in Figure 2. As can be observed in Figure 3, the PGS/MIGS scheme significantly outperforms the PGS scheme when the interference level is high. Interestingly, the sum-rate can be achieved by either κ=0 or κ=1 and no optimization over κ is required for maximizing the sum-rate. Note that, for symmetric channels, both users can achieve the same ergodic rate in the PGS/MIGS scheme by letting each user be the MIGS user 50% of the time. Hence, the MIGS scheme with switching between proper and maximally improper users can substantially improve the performance of the 2-user IC. This scheme requires as little cooperation as possible between users and further it requires no optimization or power control at the transmitter side.

## 5. Conclusions

This paper studied the ergodic rate of a PGS/IGS scheme in a 2-user Rayleigh IC treating interference as noise. Under the assumption that the users have perfect CSIR but they have access only to statistical CSIT, we derived closed-form expressions for the ergodic rates and characterized the boundary of the achievable ergodic rate region. We also proposed a practical IGS scheme which does not require any optimization and power control at the transmitter side. In this scheme, both users transmit with maximum power. One user employs MIGS while the other user employs PGS. By numerical examples, we showed that IGS can substantially increase the sum-rate and enlarge the ergodic rate region of the 2-user Rayleigh SISO-IC under strong interference.

### Future Studies

This paper, to the best of our knowledge, is the first work on IGS in IC without instantaneous CSIT, which can open a path for further studies on IGS with more practical assumptions when instantaneous CSIT is not available. It has been shown that IGS can improve the performance of multi-user (more than two users) and/or multiple antenna systems in the presence of perfect instantaneous CSIT [3,5,9,17]. However, perfect and instantaneous CSIT might not be available in practical scenarios. Thus, the following question arises, which has to be answered in future works. *Does IGS provide some gains, in multi-user (large scale scenarios) and/or multiple antenna systems when instantaneous CSIT is not available?*

## Appendix A. Proof of Lemma 1

By definition, the CDF of *Z* is

(A1) $$\begin{array}{c} F_{Z}\left(z\right)=Pr\left(Z\leq z\right)=1-Pr\left(\beta X\geq z\left(1+\alpha Y\right)\right)=\;\int_{0}^{\infty }\;\;\frac{e^{-y/\mathrm{inr}}}{\mathrm{inr}}\;\int_{\frac{z(1+\alpha y)}{\beta }}^{\infty }\;\;\frac{e^{-x/\mathrm{snr}}}{\mathrm{snr}}dxdy=1-\frac{e^{-\frac{z}{\beta \mathrm{snr}}}}{1+\frac{\alpha \mathrm{inr}}{\beta \mathrm{snr}}z}\;. \end{array}$$

Furthermore, since *Z* is a positive random variable, its expectation can be obtained as

(A2) $$\begin{array}{c} E\left\{Z\right\}=\int_{0}^{\infty }\left(1-F_{Z}\left(z\right)\right)dz=\left\{\begin{array}{ccc} \beta \mathrm{snr} & \mathrm{if} & \alpha =0,\\ \left(\frac{\beta \mathrm{snr}}{\alpha \mathrm{inr}}\right)e^{\frac{1}{\beta \mathrm{snr}}}E_{1}\left(\frac{1}{\beta \mathrm{snr}}\right) & \mathrm{if} & \alpha >0. \end{array}\right. \end{array}$$

It can be easily verified that $\bar{R}=e^{\frac{1}{\beta \mathrm{snr}}}E_{1}\left(\frac{1}{\beta \mathrm{snr}}\right)$ when α=0. Hence, in the following, we consider α>0. In this case, the CDF of $R=\log\left(1+Z\right)$ is

(A3) $$F_{R}\left(r\right)=1-e^{\frac{1}{\beta \mathrm{snr}}}e^{-\frac{e^{r}}{\beta \mathrm{snr}}}\left(\frac{1}{1-\frac{\alpha \mathrm{inr}}{\beta \mathrm{snr}}+\frac{\alpha \mathrm{inr}}{\beta \mathrm{snr}}e^{r}}\right),\;r\geq 0.$$

Since *R* is a positive random variable, its expectation may be found from its CDF as

(A4) $$E\left\{R\right\}=\int_{0}^{\infty }\left(1-F_{R}\left(r\right)\right)dr.$$

Making the change of variables $e^{r}=1+z$ in Equation (A4), $\bar{R}$ is given by the integral

(A5) $$E\left\{R\right\}=\int_{0}^{\infty }\frac{e^{-\frac{z}{\beta \mathrm{snr}}}}{1+\left(\frac{\alpha \mathrm{inr}}{\beta \mathrm{snr}}+1\right)z+\left(\frac{\alpha \mathrm{inr}}{\beta \mathrm{snr}}\right)z^{2}}dz.$$

If βsnr≠αinr, the integrand in Equation (A5) has two simple poles at z=−1 and z=−βsnr/αinr. Decomposing the integrand into simple fractions we arrive, after some change of variables, at

(A6) $$\bar{R}=\;\left(\frac{\beta \mathrm{snr}}{\beta \mathrm{snr}\;-\;\alpha \mathrm{inr}}\right)\;\;\left[\;e^{\frac{1}{\beta \mathrm{snr}}}E_{1}\left(\frac{1}{\beta \mathrm{snr}}\right)\;-e^{\frac{1}{\alpha \mathrm{inr}}}E_{1}\left(\frac{1}{\alpha \mathrm{inr}}\right)\right]\;.$$

On the other hand, if βsnr=αinr, the integrand in Equation (A5) has a double pole at z=−1. Then, Equation (A5) becomes

(A7) $$\begin{array}{c} \bar{R}=\;\;\int_{0}^{\infty }\;\frac{e^{-\frac{z}{\beta \mathrm{snr}}}}{1+z^{2}}dz=e^{\frac{1}{\beta \mathrm{snr}}}\;\;\int_{\frac{1}{\beta \mathrm{snr}}}^{\infty }\;\;\frac{e^{-t}}{t^{2}}dt=e^{\frac{1}{\beta \mathrm{snr}}}E_{2}\left(\frac{1}{\beta \mathrm{snr}}\right)\;, \end{array}$$

thus proving Lemma 1.
